# Thromboembolic Events Burden in Patients With Solid Tumors and Their Predisposing Factors

**DOI:** 10.7759/cureus.23624

**Published:** 2022-03-29

**Authors:** Shouki Bazarbashi, Turkiah Alkhaldi, Mohamed Aseafan, Maryam Melaibari, Sara Almuhisen, Samar Alharbi, Abdulrahman Alghabban, Jihad Aljumaa, Abdelmoneim Eldali, Fatma Maraiki, Tarek Owaidah, Hazzaa Alzahrani

**Affiliations:** 1 Oncology, King Faisal Specialist Hospital and Research Centre, Riyadh, SAU; 2 Pharmacy services, King Fahad Medical City, Riyadh, SAU; 3 Internal Medicine, Security Forces Hospital Program, Riyadh, SAU; 4 Pharmacology, King Abdulaziz University, Riyadh, SAU; 5 Pharmacy Services, King Fahad Medical City, Riyadh, SAU; 6 Pharmaceutical Care, King Faisal Specialist Hospital and Research Centre, Riyadh, SAU; 7 Medicine, Alfaisal University, Riyadh, SAU; 8 Biostatistics, Epidemiology, and Scientific Computing Research, King Faisal Specialist Hospital and Research Centre, Riyadh, SAU; 9 Hematology and Hematopathology, King Faisal Specialist Hospital and Research Center, Riyadh, SAU; 10 Hematology, King Faisal Specialist Hospital and Research Centre, Riyadh, SAU

**Keywords:** central venous catheter, anti-neoplastic therapy, khorana score, cancer, thromboembolism

## Abstract

Introduction: The relationship between cancers and thromboembolic events is well established. In our study, we aim to determine the burden of thromboembolic events in patients with solid tumors and identify the risk factors related to their development.

Materials & Methods: Data on patients with solid tumors and thromboembolism between January 2013 and September 2014 were collected and analyzed.

Results: During the study period 174 patients were identified. Of which, 172 (98.9%) had venous thrombus embolism, 137 (79%) were diagnosed with deep vein thromboses, 67 (38.5%) with pulmonary embolism, 84 (48.3%) were symptomatic and 90 (51.7) were incidental at diagnosis. The most common patients and disease characteristics were female sex, high body mass index (BMI), metastatic stage, colorectal and breast primaries, and anti-neoplastic therapy.

Conclusion: Our study confirmed the high burden of thromboembolic events in cancer patients and the relevant factors associated with its development.

## Introduction

The relationship between cancers and thromboembolic events (TEEs), including venous thromboembolism (VTEs) and arterial thromboembolism (ATEs), is well-known and well-established [1-3]. Evidence has shown that cancer patients have a higher risk of developing TEEs in comparison with non-cancer patients [3]. This increased risk results in an overall incidence of 1% to 43% in various cancers [4]. Venous thromboembolisms account for the majority (70% to 90%) of cases while ATEs account for only 10% to 30% [2]. Many studies have suggested that the incidence of TEEs varies according to ethnic background. Some western studies have reported that African Americans and Caucasians have a higher incidence in comparison with Hispanics and Native Americans [5]. On the other hand, most Asian studies have suggested a lower incidence of VTEs in Asian cancer patients as compared to western studies [6-9].

Along with typical TEE risk factors such as obesity, advanced age, and prolonged immobilization, cancer patients have additional risks that influence the development of TEEs. The type of malignancy, type of anti-neoplastic therapy, and presence of metastases have been considered major risk factors in cancer patients [10,11]. Many studies have shown that VTEs occur at a higher frequency in patients with cancers of the pancreas, ovaries, lung, gastric, and kidneys [4,12,13]. Certain anti-neoplastic medications also have a notably higher risk of causing TEEs. Moore et al. noted that in their study, 18.1% of patients who received cisplatin developed either VTEs or ATEs [13]. Patients receiving chemotherapy regimens containing gemcitabine also were found to be at a higher risk of developing VTEs and ATEs than those receiving non-gemcitabine-containing regimens [14]. Hormonal agents such as tamoxifen, newer anti-neoplastic agents such as anti-angiogenic agents, and anti-epidermal growth factor receptor antibodies have all been shown to increase the risk of VTEs and ATEs in cancer patients in several studies [15-18]. Additionally, patients with metastatic diseases were found to be at a higher risk for VTEs, the risk ranging from 1.4 to 21.5-fold, compared to patients with limited disease [5,19].

Established guidelines recommend primary prophylaxis for all hospitalized cancer patients, including those with reduced mobility and central venous catheters [20]. Routine pharmacologic thromboprophylaxis for outpatient cancer patients is still controversial and not widely recommended [21,22]. However, recent guidelines by scientific societies have recommended thromboprophylaxis for patients with cancer and high risk for VTE [20]. Several attempts have been made to establish a scoring system based on known risk factors to identify cancer patients at a higher risk for VTEs. The most popular are the Khorana model, the Vienna modification of the Khorana model, the PROTECHT scoring system, the CONKO score, and the most recently described but not validated COMPASS-CAT scoring system [21,23-27]. Whether thromboprophylaxis results in a significant reduction in death due to VTEs among high-risk groups remains to be established.

The primary objective of this study was to determine the incidence of different types of TEEs in patients with solid tumors and established thromboembolism diagnoses at a single institution in the Middle East. In addition, the study identified the patients and disease characteristics related to thrombosis events.

## Materials and methods

Study design

This was a retrospective single-institution study conducted at a national referral center for cancer in Saudi Arabia. Adult patients (≥18 years) with solid tumor diagnoses who had developed TEEs in either the inpatient or outpatient setting between January 2013 and September 2014 were eligible for inclusion. Patients were included regardless of their cancer stage at diagnosis, on active therapy or surveillance, in remission or with active disease. Patients with prior history of TEE who had developed new TEE during the above period were also included in the study. Patients with no history of cancer diagnosis and/or no TEE during the above period were not eligible for the study. 

Data collection

The medical records of the eligible patients were reviewed for the following information: patient-related factors including age, sex, body mass index, co-morbid conditions, and Eastern Cooperative Oncology Group (ECOG) performance status; cancer-related factors such as the primary tumor site and disease stage; treatment-related factors including anti-neoplastic agents and radiotherapy in use at the time of the TEE; recent surgical procedures; and blood transfusions. Finally, TEE-related factors, including the types (venous versus arterial), were reviewed. Other information such as diagnosis method, clinical presentation, previous TEE history, history of current VTE prophylaxis, hospitalization at the time of TEE, relationship to the central venous catheters, and finally, the Khorana score.

The study was conducted as per the ethical principles contained in the declaration of Helsinki (2000), Good Clinical Practice Guidelines and the policies and guidelines of the institution it was carried in. The study was approved by the institutional review board of King Faisal Specialist Hospital and Research Centre, Riyadh, SAU (approval no 2141138). Given the retrospective nature of the study, a waiver of consent was obtained from the institutional review board. The identity of the patients studied remained anonymous since no identifying data or protected health information were recorded. All data were password secured to safeguard the confidentiality of the collected data.

Statistical analysis

This was a descriptive study. Statistical Package for Social Sciences (SPSS) software version 20 (IBM Corp., Armonk, NY, USA) was used to analyze the data. Continuous variables were described as mean ± standard deviation, categorical variables were described as numbers (percentages), and cross-tabulation was used to summarize the relationships between two categorical variables. 

## Results

A total of 312 medical records were reviewed, and 174 of them met the eligibility criteria. Reasons for non-eligibility was nonmalignant diagnosis or less than 18 years of age. The most common thromboembolism (98.9%) was venous TEE, as seen in Table 1. The venous TEEs were most commonly presented as peripheral deep venous thrombosis (DVT) (50%), which combined upper and lower DVT, and pulmonary embolism (PE) (38.5%). Others were visceral veins thrombosis (17.2%), jugular thrombosis (8%), and renal vein thrombosis (3.4%). Around 48.3% of the patients were symptomatic, and 51.7% were diagnosed incidentally. The most common diagnostic method was a CT scan (69%). About 31% of patients were admitted for management of their TEEs. Factors such as the previous incidence of TEE at diagnosis and relation to central venous catheter were low (8.6% and 13.8%, respectively). Twenty-three percent of patients were diagnosed with TEE during hospitalization. The majority of patients had either an intermediate or high Khorana score-developed mainly for outpatient settings-of 73% with only 10.3% of them on a DVT prophylaxis treatment at the time of TEE diagnosis.

**Table 1:** Incidence, characteristics, and risk factors related to 174 TEE events TEEs: Thromboembolic events, PE: Pulmonary embolism, DVT: Deep vine thrombosis, VTE: Venous thromboembolism

Item	No (%)
Type of TEE^a^	
Arterial	2 (1.1)
Venous	172 (98.9)
PE^b^	67 (38.5)
DVT^c^	87 (50)
Lower extremities DVT	44 (25.3)
Upper extremities DVT	29 (16.7)
Other venous thromboses	
Jugular	14 (8)
Renal vein thrombosis	6 (3.4)
Visceral veins thrombosis	30 (17.2)
Clinical presentation	
Symptomatic	84 (48.3)
Incidental	90 (51.7)
Diagnostic method	
Computerized tomography	120 (69)
Doppler ultrasound	52 (29.9)
Computerized tomography angiography	2 (1.1)
History of TEEs	15 (8.6)
Current hospitalization at TEE	40 (23)
Admission because of TEE	54 (31)
Relation to central venous catheter	24 (13.8)
Current VTE^d^ prophylaxis at diagnosis	18 (10.3)
Khorana Score	
Low	37 (21.3)
Intermediate	93 (53.5)
High	34 (19.5)
Not identified	10 (5.7)

Baseline patients, disease, and treatment characteristics are shown in Table 2. The majority of patients were females (69%) and had an ECOG performance status of 2 or higher (47.7%). The mean body mass index (BMI) was 28 kg/m2. Hypertension (27%) and diabetes mellitus (21.3%) were the most common associated co-morbidities. Colorectal cancer represented the largest group of cancer patients (19%), followed by breast cancer (18.4%) and lymphoma (10.9%); an almost similar number occurred among ovarian, stomach, ampulla of vater, and lung cancers (~5-7%). Most of the TEE cases were found in patients with metastatic disease (71.8%). Of the 125 patients with metastatic disease, 80 (64%) received anti-neoplastic therapy (targeted, hormonal, and/or chemotherapy), with a similar percentage for non-metastatic diseases. In all patient groups, 62% of patients were on chemotherapy within two months before their TEE diagnosis. Other treatment-related known risk factors such as surgery, blood transfusion, and radiotherapy were low (< 20%). The most common chemotherapy agents were capecitabine and oxaliplatin (16.1%, and 15.5%, respectively), which is in accordance with the most common cancer diagnosis i.e., colorectal cancer.

**Table 2:** Baseline patients, disease, and treatment characteristics of 174 subjects with TEE BMI: Body mass index, SD: Standard deviation, ECOG: Eastern Cooperative Oncology Group

Item	No (%)
Age, median (range)	50 ± 16.5
Sex	
Male	54 (31)
Female	120 (69)
BMI^a^ (mean + SD^b^)	28 ± 6
Co-morbidities	
Hypertension	47 (27)
Diabetes mellitus	37 (21.3)
Lung disease	13 (7.5)
Atrial fibrillation/flutter	5 (2.9)
Heart failure	3 (1.7)
Liver disease	2 (1.1)
Others	4 (25.3)
Performance status (ECOG^c^)	
0	10 (5.7)
1	53 (30.5)
2	40 (23)
3	35 (20.1)
4	8 (4.6)
Unknown	28 (16.1)
Primary malignancy	
Colorectal	33 (19)
Breast	32 (18.4)
Lymphoma	19 (10.9)
Ovary	13 (7.6)
Stomach	12 (6.9)
Ampulla of vater	12 (6.9)
Lung	11 (6.3)
Pancreas	8 (4.6)
Corpus uteri	5 (2.9)
Head and neck	3 (1.7)
Liver	2 (1.1)
Cervix uteri	2 (1.1)
Sarcoma	2 (1.1)
Esophagus	1 (0.6)
Others	19 (10.9)
Cancer stage	
Localized	22 (12.6)
Regional	27 (15.6)
Metastatic	125 (71.8)
Treatment received within 60 days	
Major surgery	22 (12.6)
Blood transfusion	31 (17.8)
Radiotherapy	11 (6.3)
Targeted therapy	27 (15.5)
Hormonal therapy	8 (4.6)
Chemotherapy	108 (62)
Capecitabine	28 (16.1)
Oxaliplatin	27 (15.5)
Cyclophosphamide	17 (9.8)
5-Fluorouracil	16 (9.2)
Doxorubicin	15 (8.6)
Cisplatin	12 (6.9)
Carboplatin	11 (6.3)
Vincristine	10 (5.7)
Gemcitabine	7 (4)
6-Mercaptopurine	2 (1.1)
Cytarabine	1 (0.6)
Others	34 (19.5)
Not on treatment	44 (25)

## Discussion

Our study represents an evaluation of all TEEs diagnosed in patients with solid tumors within two years at a single institution. The study showed a majority of venous TEEs, with the most common being DVT (50%) and PE (38.5%). Incidental diagnoses and symptomatic diseases were similar (~50% each), with 31% requiring hospitalization. Khorana score is a risk assessment model based on five clinical and laboratory parameters that were validated to assess the risk for cancer associated VTE in the outpatient setting [22]. The most prevalent Khorana scores were intermediate and high (77.4%) in this population. The most common patient and disease characteristics identified in the study included female sex, elevated BMI, hypertension and diabetes co-morbidities, metastatic stage, colorectal and breast cancer sites, and anti-neoplastic therapy administration within the last two months of presentation.

The study highlights several aspects, some of which are controversial, related to TEEs in cancer patients. One of these is sex. Many reports have suggested that TEEs are more common in females [12,27]; others, however, have not confirmed this finding [28-30]. In our study, the majority of our subjects who had TEEs (69.0%) were females. This might be related to the higher prevalence of breast and ovarian cancer in our patient population. Certain comorbidities (cardiovascular, hypertension, diabetes, and obesity) have been shown to considerably increase the risk for TEE development [31]. In our study, 47 (27.0%) and 37 (21.3%) of the patients had hypertension and diabetes, respectively. These numbers are not different from the incidence of diabetes and hypertension in the general population in our region [32,33] and do not support this hypothesis.

Colorectal cancer was the most common cancer seen in our study, with 33 patients representing 19% of our patient population. This finding is not consistent with many other studies [5,13,34] and might indicate a higher risk for TEEs in our colorectal patients, which represents less frequent cancer than breast cancer, seen at our institution [35]. Breast cancer represents a low-risk type of malignancy in most risk models, such as the Khorana score [23,27]; however, it was the second most common malignancy (18.4%) with TEEs in our patient population.

We also looked at other reported risk factors that affect the incidence of TEEs in cancer patients. Performance status is one of them. Previous reports have demonstrated that poor performance status is an important risk factor for VTEs in cancer patients [34,36]. In our study, patients with ECOG performance status scores of 2 or more had the highest frequency (47.7%) of TEEs. However, the percentage of patients with ECOG performance status 1 was still relatively high (30.5%). Advanced stage and anti-neoplastic therapy are other important identified risk factors [13,37,38]. In our study, 71.8% of patients had metastatic disease, 75% were on anti-neoplastic therapy, and 65.5% had metastatic disease and were on anti-neoplastic therapy. Of note, capecitabine and oxaliplatin were the most common agents used in medications in our cohort of patients. Epirubicin, oxaliplatin, and capecitabine (EOX) regimens had been implicated with an increased incidence of TEEs in gastric and esophageal cancer patients receiving pre-operative or peri-operative chemotherapy [39,40]. Most of the data related to capecitabine and oxaliplatin in colorectal cancer were, however, coupled with bevacizumab, which has thromboembolic properties in itself [41,42]. Twenty-four (13.8%) of the TEEs were catheter-related. Catheter-related thrombosis was low but more common than expected. Historically, catheter-related TEEs are more likely to be diagnosed incidentally and to occur in PICC lines than implantable catheters [43,44]. Hospitalization is also an important risk factor for TEEs in general [45], and more so for cancer-associated TEEs [32]. This risk factor did represent one-fourth (23%) of all TEEs diagnosed in our study group.

Thromboembolic events in cancer patients adversely affect survival whether discovered symptomatically or incidentally through routine imaging [46,47]. In our study, nearly half of the TEE cases presented with incidental VTE. These findings were supported by other studies showing that incidental VTE accounts for half of the TEE cases [48,49]. In addition to affecting survival, TEE in cancer patients places a humanistic and economic burden on patients and institutions [50]. In our study, around one-third (31%) of our patients had to be admitted for their TEEs. This article was previously posted to the Research Square preprint server on May 6th, 2021.

Several attempts have been made to identify patients at high risk of developing TEEs by developing risk assessment models. The most used model has been the Khorana scoring system [23]. Many other risk assessment models have also been established [27,29,32]. The majority of the patients in our study fell in the intermediate and high-risk score for the Khorana model, constituting 77.4%. Recently, two trials tested the efficacy of oral anticoagulants in the prevention of venous thromboembolic events in patients with intermediate or high-risk Khorana scores. In the CASSINI trial, rivaroxaban significantly reduced the number of VTEs and VTE-related deaths during the on-treatment period [50]. Similarly, in the AVERT trial, apixaban resulted in a significantly lower rate of VTE than the placebo in patients with intermediate and high-risk Khorana Scores [51].

Our study has several limitations. First, it is a single institution and a retrospective study. Additionally, the study does not calculate the incidence of TEE in our patient population.

## Conclusions

Our study confirms the high burden of TEE in cancer patients. It also highlights several factors, including advanced cancer stage, cancer site, anti-neoplastic therapy, central venous catheter, and the role of certain chemotherapy agents in the development of TEEs in cancer patients. It also supports the importance of risk assessment models in identifying cancer patients at risk of TEE, and the need for future studies to establish treatment prevention in those patients with intermediate and high Khorana scores.

## References

[1] Varki A (2007). Trousseau's syndrome: multiple definitions and multiple mechanisms. Blood.

[2] Sutherland DE, Weitz IC, Liebman HA (2003). Thromboembolic complications of cancer: epidemiology, pathogenesis, diagnosis, and treatment. Am J Hematol.

[3] Levitan N, Dowlati A, Remick SC (1999). Rates of initial and recurrent thromboembolic disease among patients with malignancy versus those without malignancy. Risk analysis using Medicare claims data. Medicine.

[4] Lee AY, Levine MN (2003). Venous thromboembolism and cancer: risks and outcomes. Circulation.

[5] Amer MH (2013). Cancer-associated thrombosis: clinical presentation and survival. Cancer Manag Res.

[6] Oh SY, Kim JH, Lee KW, Bang SM, Hwang JH, Oh D, Lee JS (2008). Venous thromboembolism in patients with pancreatic adenocarcinoma: lower incidence in Asian ethnicity. Thromb Res.

[7] Kang MJ, Ryoo BY, Ryu MH (2012). Venous thromboembolism (VTE) in patients with advanced gastric cancer: an Asian experience. Eur J Cancer.

[8] Kim JW, Chun EJ, Choi SI (2013). A prospective study on the incidence of postoperative venous thromboembolism in Korean gastric cancer patients: an inquiry into the application of Western guidelines to Asian cancer patients. PLoS One.

[9] Huang H, Korn JR, Mallick R, Friedman M, Nichols C, Menzin J (2012). Incidence of venous thromboembolism among chemotherapy-treated patients with lung cancer and its association with mortality: a retrospective database study. J Thromb Thrombolysis.

[10] Stricker H (2014). Venous thromboembolism and cancer: pathophysiology and incidence. Vasa.

[11] Horsted F, West J, Grainge MJ (2012). Risk of venous thromboembolism in patients with cancer: a systematic review and meta-analysis. PLoS Med.

[12] Streiff MB (2013). Association between cancer types, cancer treatments, and venous thromboembolism in medical oncology patients. Clin Adv Hematol Oncol.

[13] Moore RA, Adel N, Riedel E (2011). High incidence of thromboembolic events in patients treated with cisplatin-based chemotherapy: a large retrospective analysis. J Clin Oncol.

[14] Qi WX, Lin F, Sun YJ, Tang LN, Shen Z, Yao Y (2013). Risk of venous and arterial thromboembolic events in cancer patients treated with gemcitabine: a systematic review and meta-analysis. Br J Clin Pharmacol.

[15] Hernandez RK, Sørensen HT, Pedersen L, Jacobsen J, Lash TL (2009). Tamoxifen treatment and risk of deep venous thrombosis and pulmonary embolism: a Danish population-based cohort study. Cancer.

[16] Choueiri TK, Schutz FA, Je Y, Rosenberg JE, Bellmunt J (2010). Risk of arterial thromboembolic events with sunitinib and sorafenib: a systematic review and meta-analysis of clinical trials. J Clin Oncol.

[17] Ranpura V, Hapani S, Chuang J, Wu S (2010). Risk of cardiac ischemia and arterial thromboembolic events with the angiogenesis inhibitor bevacizumab in cancer patients: a meta-analysis of randomized controlled trials. Acta Oncol.

[18] Petrelli F, Cabiddu M, Borgonovo K, Barni S (2012). Risk of venous and arterial thromboembolic events associated with anti-EGFR agents: a meta-analysis of randomized clinical trials. Ann Oncol.

[19] Konigsbrugge O, Pabinger I, Ay C (2014). Risk factors for venous thromboembolism in cancer: novel findings from the Vienna Cancer and Thrombosis Study (CATS). Thromb Res.

[20] Lyman GH, Khorana AA, Kuderer NM (2013). Venous thromboembolism prophylaxis and treatment in patients with cancer: American Society of Clinical Oncology clinical practice guideline update. J Clin Oncol.

[21] Guyatt GH, Akl EA, Crowther M, Gutterman DD, Schuünemann HJ (2012). Executive summary: antithrombotic therapy and prevention of thrombosis, 9th ed: American College of Chest Physicians Evidence-Based Clinical Practice Guidelines. Chest.

[22] Khorana AA, Kuderer NM, Culakova E, Lyman GH, Francis CW (2008). Development and validation of a predictive model for chemotherapy-associated thrombosis. Blood.

[23] Ay C, Dunkler D, Marosi C (2010). Prediction of venous thromboembolism in cancer patients. Blood.

[24] Verso M, Agnelli G, Barni S, Gasparini G, LaBianca R (2012). A modified Khorana risk assessment score for venous thromboembolism in cancer patients receiving chemotherapy: the Protecht score. Intern Emerg Med.

[25] Pelzer U, Sinn M, Stieler J, Riess H (2013). Primary pharmacological prevention of thromboembolic events in ambulatory patients with advanced pancreatic cancer treated with chemotherapy?. Dtsch Med Wochenschr.

[26] Pabinger I, van Es N, Heinze G (2018). A clinical prediction model for cancer-associated venous thromboembolism: a development and validation study in two independent prospective cohorts. Lancet Haematol.

[27] Khorana AA, Francis CW, Culakova E, Kuderer NM, Lyman GH (2007). Frequency, risk factors, and trends for venous thromboembolism among hospitalized cancer patients. Cancer.

[28] Blom JW, Doggen CJ, Osanto S, Rosendaal FR (2005). Malignancies, prothrombotic mutations, and the risk of venous thrombosis. JAMA.

[29] Farge D, Bounameaux H, Bauersachs RM, Brenner B (2017). Women, thrombosis, and cancer: a gender-specific analysis. Thromb Res.

[30] Chew HK, Wun T, Harvey D, Zhou H, White RH (2006). Incidence of venous thromboembolism and its effect on survival among patients with common cancers. Arch Intern Med.

[31] Gerotziafas GT, Taher A, Abdel-Razeq H (2017). A predictive score for thrombosis associated with breast, colorectal, lung, or ovarian cancer: the prospective COMPASS-cancer-associated thrombosis study. Oncologist.

[32] Meo SA (2016). Prevalence and future prediction of type 2 diabetes mellitus in the Kingdom of Saudi Arabia: a systematic review of published studies. J Pak Med Assoc.

[33] Yoon SS, Carroll MD, Fryar CD (2015). Hypertension prevalence and control among adults: United States. NCHS.

[34] Al Diab AI (2010). Cancer-related venous thromboembolism: insight into underestimated risk factors. Hematol Oncol Stem Cell Ther.

[35] (2018). King Faisal Specialist Hospital and Research Center cancer registry report. (2014).. https://www.kfshrc.edu.sa/store/media/8el.pdf.

[36] Pabinger I, Ay C (2012). Risk of venous thromboembolism and primary prophylaxis in cancer. Should all patients receive thromboprophylaxis?. Hamostaseologie.

[37] Blom JW, Vanderschoot JP, Oostindiër MJ, Osanto S, van der Meer FJ, Rosendaal FR (2006). Incidence of venous thrombosis in a large cohort of 66,329 cancer patients: results of a record linkage study. J Thromb Haemost.

[38] Papaxoinis G, Kamposioras K, Germetaki T (2018). Predictive factors of thromboembolic complications in patients with esophagogatric adenocarcinoma undergoing preoperative chemotherapy. Acta Oncol.

[39] Khanna A, Reece-Smith AM, Cunnell M, Madhusudan S, Thomas A, Bowrey DJ, Parsons SL (2014). Venous thromboembolism in patients receiving perioperative chemotherapy for esophagogastric cancer. Dis Esophagus.

[40] Van Cutsem E, Rivera F, Berry S (2009). Safety and efficacy of first-line bevacizumab with FOLFOX, XELOX, FOLFIRI and fluoropyrimidines in metastatic colorectal cancer: the BEAT study. Ann Oncol.

[41] Hong YS, Lee J, Kim KP (2013). Multicenter phase II study of second-line bevacizumab plus doublet combination chemotherapy in patients with metastatic colorectal cancer progressed after upfront bevacizumab plus doublet combination chemotherapy. Invest New Drugs.

[42] Yukisawa S, Fujiwara Y, Yamamoto Y, Ueno T, Matsueda K, Kohno A, Suenaga M (2010). Upper-extremity deep vein thrombosis related to central venous port systems implanted in cancer patients. Br J Radiol.

[43] Saber W, Moua T, Williams EC (2011). Risk factors for catheter-related thrombosis (CRT) in cancer patients: a patient-level data (IPD) meta-analysis of clinical trials and prospective studies. J Thromb Haemost.

[44] Tagalakis V, Patenaude V, Kahn SR, Suissa S (2013). Incidence of and mortality from venous thromboembolism in a real-world population: the Q-VTE study cohort. Am J Med.

[45] O'Connell C, Razavi P, Ghalichi M (2011). Unsuspected pulmonary emboli adversely impact survival in patients with cancer undergoing routine staging multi-row detector computed tomography scanning. J Thromb Haemost.

[46] Connolly GC, Menapace L, Safadjou S, Francis CW, Khorana AA (2013). Prevalence and clinical significance of incidental and clinically suspected venous thromboembolism in lung cancer patients. Clin Lung Cancer.

[47] van Es N, Bleker SM, Di Nisio M (2014). Cancer-associated unsuspected pulmonary embolism. Thromb Res.

[48] Dentali F, Ageno W, Becattini C (2010). Prevalence and clinical history of incidental, asymptomatic pulmonary embolism: a meta-analysis. Thromb Res.

[49] Kourlaba G, Relakis J, Mylonas C, Kapaki V, Kontodimas S, Holm MV, Maniadakis N (2015). The humanistic and economic burden of venous thromboembolism in cancer patients: a systematic review. Blood Coagul Fibrinolysis.

[50] Khorana AA, Soff GA, Kakkar AK (2019). Rivaroxaban for thromboprophylaxis in high-risk ambulatory patients with cancer. N Engl J Med.

[51] Carrier M, Abou-Nassar K, Mallick R (2019). Apixaban to prevent venous thromboembolism in patients with cancer. N Engl J Med.

